# Clinical Approaches to Late-Onset Psychosis

**DOI:** 10.3390/jpm12030381

**Published:** 2022-03-02

**Authors:** Kiwon Kim, Hong Jin Jeon, Woojae Myung, Seung Wan Suh, Su Jeong Seong, Jae Yeon Hwang, Je il Ryu, Seon-Cheol Park

**Affiliations:** 1 Department of Psychiatry, Kangdong Sacred Heart Hospital, College of Medicine, Hallym University, Seoul 05355, Korea; kkewni@gmail.com (K.K.); swsuh01@hanmail.net (S.W.S.); seongsj01@gmail.com (S.J.S.); hjaeyeon@gmail.com (J.Y.H.); 2 Department of Psychiatry, Depression Center, Samsung Medical Center, School of Medicine, Sungkyunkwan University, Irwon-ro, Gangnam-gu, Seoul 06351, Korea; jeonhj@skku.edu; 3 Department of Neuropsychiatry, Seoul National University Bundang Hospital, Gumi-ro, 173 beon-gil Bundang-gu, Seongnam-si 13619, Korea; wmyung@snu.ac.kr; 4 Department of Neurosurgery, College of Medicine, Hanyang University, Gyungchun-ro 153, Guri-si 11923, Korea; 5 Department of Neurosurgery, Hanyang University Guri Hospital, Gyungchun-ro 153, Guri-si 11923, Korea; 6 Department of Psychiatry, College of Medicine, Hanyang University, Gyungchun-ro 153, Guri-si 11923, Korea; 7 Department of Psychiatry, Hanyang University Guri Hospital, Gyungchun-ro 153, Guri-si 11923, Korea

**Keywords:** late-onset schizophrenia-like psychosis, secondary psychosis, management

## Abstract

Psychosis can include schizophrenia, mood disorders with psychotic features, delusional disorder, active delirium, and neurodegenerative disorders accompanied by various psychotic symptoms. Late-onset psychosis requires careful intervention due to the greater associated risks of secondary psychosis; higher morbidity and mortality rates than early-onset psychosis; and complicated treatment considerations due to the higher incidence of adverse effects, even with the black box warning against antipsychotics. Pharmacological treatment, including antipsychotics, should be carefully initiated with the lowest dosage for short-term efficacy and monitoring of adverse side effects. Further research involving larger samples, more trials with different countries working in consortia, and unified operational definitions for diagnosis will help elaborate the clinical characteristics of late-onset psychosis and lead to the development of treatment approaches.

## 1. Introduction

The definition of psychosis encompasses the presence of delusions, hallucinations, or both, without insights into the condition of others, which are derived from the problem of reality testing [1,2]. Psychosis may cover diagnoses such as schizophrenia, mood disorder with psychotic features, delusional disorders, delirium of an active type, and neurodegenerative disorders accompanied by various psychotic symptoms. The definition of late-onset psychosis is not intended to merely discriminate it from early-onset psychosis based on differences between their neuropsychopathologies; it is also intended to help individualize management carefully with older age. 

Late-onset psychosis requires careful intervention for several reasons, including higher risks of secondary psychosis in older adults, higher morbidity and mortality rates than early-onset psychosis, and complicated treatment considerations due to a higher incidence of adverse effects [3,4]. The clinical considerations of secondary psychosis in older adults are important for its preponderance, leading to 60% of the etiologies of late-onset psychosis. It is necessary to consider differential diagnoses, including neurodegenerative disorders, characterized by delirium and substance-induced psychosis, and the effects of prescribed medications and illicit drugs [5,6].

Here, we will investigate the overall epidemiology, clinical characteristics, recent research findings on neuropsychological characteristics, and treatment approaches with the careful management of side effects with a general focus on late-onset psychosis and a more specific focus on late-onset schizophrenia such as psychosis. Because of the complexity of psychotic symptoms associated with neurodegenerative disorders, so-called dementia, we will exclude further discussion on the behavioral and psychological symptoms of dementia in this review.

At the end of the discussion, we will outline a few suggestions for future research to investigate late-onset psychosis. 

## 2. History and Epidemiology

Regarding the narrowest concept of late-onset psychosis, as late-onset schizophrenia, Manfred Bleuler first observed patients whose symptoms started after 40 years but found that 50% of them reported symptoms similar to those of early-onset schizophrenia [7]. Despite the poor discrimination, this concept led the German literature to use a cut-off age of 40 years, leading to setting the onset of late paraphrenia at 55–60 years. Late paraphrenia was applied to patients with chronic schizophrenia, but it had similar psychotic symptoms without obvious functional deterioration. These patients were thought to experience hallucinations and delusions but had no deterioration or had scarce affective changes, which were different from dementia praecox, which was named by Kraepelin [8]. With the increasing clinical interest in geriatric psychiatry, the clinical characteristics differentiating early-onset and late-onset schizophrenia were reported more and more and needed further elaboration. There was a lack of information and findings, including the differences and common features of early-onset and late-onset schizophrenia; early versions of psychiatric diagnostic systems did not specify the age limit in the criteria for schizophrenia. Currently, the diagnostic criteria for schizophrenia include those with onset after the age of 45 years, followed by specifiers for late-onset with a demarcation at 45 years. The age-related criteria or specifiers have finally been removed [8]. There are no distinguished systematic diagnostic criteria for late-onset schizophrenia; however, the International Late-Onset Schizophrenia Group (ILOSG) have formulated specific definitions and research questions for this issue. They proposed the terms with ‘Late-Onset Schizophrenia’ and ‘Very Late-Onset Schizophrenia-Like Psychosis’ for disorders with onset between 40 to 60 years and after the age of 60, respectively [8,9]. 

Schizophrenia is reported to have an overall lifetime prevalence of 1% but a lower prevalence among older adults (0.1–0.5%) [10]. As mentioned before, patients with schizophrenia can be divided into three groups with the effort of ILOSG: early-onset with onset before 40 years, late-onset with onsets within 40–60 years, and very late-onset with onset after 60 years. A previous report observed that approximately 75–80% of individuals with schizophrenia have early onsets; those with late or very late onsets account for 20–25% [11]. Epidemiological data on schizoaffective disorders in older adults, delusional disorders in older adults, or mood disorders with psychotic features observed in older adults are lacking because of the small sample size and scarcity. The diagnostic criteria for schizoaffective disorders are complicated; mood episodes and psychotic symptoms without mood episodes are more prevalent, and the age of onset of schizoaffective disorders is greater than that of individuals with pure schizophrenia. A study with case registration data also estimated the 1-year prevalence of schizoaffective disorders in older adults aged more than 60 years as 0.14%, which was less than the prevalence of schizophrenia in the same age group of 0.55% [12]. Estimating the prevalence of delusional disorders is also difficult because of their ambiguity and disentangled heterogeneity. The previously estimated lifetime prevalence of delusional disorders was 0.18%, including a prevalence of 0.03% for older adults, usually with an average age of onset of 48 years old [13]. The pooled incidence of affective psychosis in older adults reported in a systematic review was 30.9 per 100,000 person-years [14]. Given the increasing prevalence of psychotic depression in the aging group, this estimated prevalence may be less than the actual rate, based on the average age of onset of 51 years for psychotic depression and the higher incidence of delusion of up to 45% for older in-patients diagnosed with depression [15]. Psychotic symptoms associated with neurodegenerative disorders often have a high reported prevalence of up to 40% for Alzheimer’s disease, 25–78% for Lewy body disease, and almost 15% for vascular dementia [16]. Delirium, commonly observed in older adults, can also accompany psychotic symptoms. The estimated prevalence was 42.7% for psychotic symptoms, 27% for visual hallucinations, 12.4% for auditory hallucinations, 2.7% for tactile hallucinations, and 25.6% for delusions in patients with delirium [5]. Fluctuating orientation and changes in vigilance may be the key signs of delirium that may differentiate it from other secondary psychoses in older adults, which are difficult to distinguish due to diverse etiologies, including neurodegenerative changes, high medical comorbidity, and polypharmacy. 

## 3. Clinical Characteristics of Late-Onset Psychosis 

### 3.1. Clinical Characteristics in Late-Onset Schizophrenia 

Overall, the global psychopathologic symptoms of early-onset and late-onset schizophrenia are similar [17]. However, patients with late-onset schizophrenia tend to have more visual, olfactory, tactile, and auditory hallucinations, with more accusatory characteristics [8]. Those with late-onset schizophrenia were observed to have fewer formal thought disorders and negative symptoms but a higher prevalence of persecutory and partition delusions [18]. Female preponderance was observed in both early- and late-onset schizophrenia, a more definite report in the very late-onset schizophrenia group [8,14]. Some research groups have reported more impaired function in learning and memory retention in individuals with very late-onset schizophrenia, suggesting the possibility of an association with neurodegeneration [19]. This risk of progressive cognitive deterioration was also indicated by brain structural abnormalities [20]; however, this was not reproducible, and warrants further research. Family history related to schizophrenia was more frequently observed in early- and late-onset schizophrenia, which shows a weak genetic association in those with very late-onset schizophrenia [21]. Older age of onset was also associated with lower rates of substance use, better premorbid psychosocial function, and higher achievement during school age. Late-life schizophrenia was observed to have a greater association with psychological burdens such as unemployment [22]. Early childhood maladjustments were also observed to be associated with early- and late-onset schizophrenia but not in the very late-onset schizophrenia group. The dosage of medications for treating symptoms was significantly higher for early-onset schizophrenia but lower for late-onset and very late-onset groups [23]. The risk of tardive dyskinesia was found to be predominantly high for patients with very late-onset schizophrenia [6]. 

Overall, neuroimaging findings correlated with schizophrenia have been consistently observed in individuals with early-onset, late-onset, and very late-onset schizophrenia [24]. These findings involve volumetric atrophy, white matter hyperintensity, and poor functional connectivity related to the frontal and temporoparietal lobes. Compared to healthy controls, individuals with late-onset schizophrenia seem to have larger ventricles, which was prominent in the lateral and third ventricles [25]. They also showed smaller volumes of the hippocampus and amygdala. Other findings included a decrease in the hippocampus/amygdala ratio, which was correlated with the age of onset of schizophrenia, suggesting a neurodevelopmental signature [26]. Apart from the comparison of healthy controls and individuals with schizophrenia, a comparison of neuroimaging findings of late-onset and early-onset schizophrenia showed various findings [27]. Some researchers reported larger thalamic volumes, more atrophy in the cerebellum, and a decrease in corpus callosum volume during late-onset schizophrenia [26,28], while others found no significant difference between the two groups [29,30]. A decreased perfusion of the lower frontal and temporal lobes was observed in patients with late-onset schizophrenia compared with healthy controls and early-onset schizophrenia patients. Additionally, white matter hyperintensities, which are vascular risk factors, were more frequently observed in the temporoparietal, occipital, and thalamic regions in late-onset schizophrenia patients than in healthy controls and early-onset schizophrenia patients [31,32,33]. 

### 3.2. Clinical Characteristics of Psychotic Depression in Older Adults

The clinical differences in psychotic depression among older adults also need attention because of the tendency of greater symptom severity, greater psychic anxiety, and hypochondriasis comorbidity, which could involve somatic delusions [34]. Considering the common prevalence of psychotic depression in older adults in psychiatric wards, careful intervention with symptomatic evaluation is necessary. However, they are less likely to report delusions of guilt or paranoid delusions. Recent meta-analyses comparing studies related to psychotic depression in older adults and those related to non-psychotic depression in older adults found an elevated risk of suicide attempts in individuals with psychotic depression with odds ratios of 2.11 during their lifetime and 1.93 during the acute phase [35]. A similar meta-analysis also reported an increased risk of suicide associated with psychotic depression, emphasizing the need for clinicians to be alert for active involvement in psychotic depression management and the constitution of supreme priority on anti-suicidal interventions [36]. 

### 3.3. Clinical Characteristics in Secondary Psychosis 

Various conditions, such as traumatic brain injury; systemic diseases including autoimmune disorders, cerebrovascular diseases, malignant disorders primarily in the central nervous system or malignancy disseminated from other sites, infections, neurodegenerative disorders, and seizure disorders; and illicit or over-the-counter drugs or prescribed medications, can lead to psychotic symptoms in older adults [37]. 

Clinicians should consider differential diagnoses and look out for the following features [38]: atypical presentation of psychosis, such as a later age of onset, deviating from the typical period of onset; absence of previous psychiatric history or family history; visual hallucinations; accompanied disorientation or confusion, such as altered consciousness; the presence of other medical symptoms; temporal relationship between psychotic symptoms and known medical disorders, such as uncontrolled diabetes mellitus; and prescription or over-the-counter medications or substance abuse. 

### 3.4. Clinical Characteristics in Delirium 

Delirium is a condition characterized by the disturbance of attention and awareness within a short period, which can cause symptomatic fluctuation. Recent criteria also include a disturbance of one or more aspects of the cognitive domain, including memory, orientation, language, visuospatial ability, and perception. Individuals with delirium presenting with psychotic symptoms may also show sleep–wake cycle disruption, psychomotor disturbance of an inactive or active type, and inappropriate emotional behavior [39]. In the clinical situation, risk factors that could be associated with delirium should be considered, such as dementia, pre-existing cognitive problems, functional impairment, visual impairment, and history of other substance use. Polypharmacy, concomitant use of psychoactive drugs, physical restraints, and abnormal laboratory results with systematic problems are common precipitating factors. 

## 4. Diagnostic Considerations for Late-Onset Psychosis 

Given the previous findings related to diagnostic entities associated with psychotic symptoms in older adults, careful approaches are necessary for accurate diagnosis. Late-onset psychosis should be considered as a rule-out diagnosis, assuming that until the last period, 60% of the cases of psychosis in older adults have secondary causes [37,40]. Based on the inconsistent reports and various neuropsychological findings we should be aware that no definite signs or symptoms indicate a specific diagnosis of psychotic symptoms in older adults. Clinicians should consider several causes of late-life psychosis, from late-onset schizophrenia to psychosis secondary to substance abuse. 

A thorough history obtained from the individual suffering from psychotic symptoms and their family members is necessary. Physical examinations, including neurological examinations and cognitive assessments, would help to gather information related to overall physical states and the possibility of neurodegenerative changes. Running laboratory tests and brain imaging, such as magnetic resonance imaging (MRI) or computerized tomography (CT), may be useful to rule out structural cerebral lesions. Complete blood count, screening to evaluate liver function, renal function, electrolyte imbalance, metabolic panel, thyroid function test, vitamin B12, folic acid concentration, urine toxicology, and infectious disease markers including syphilis, HIV panels, and autoimmune panels are helpful [37] in differentiating diagnosis. Neuropsychological tests for determining the cognitive changes in individuals will also help in identifying the etiology of psychotic symptoms. 

## 5. Consideration of Treatment Approaches for Late-Life Psychosis

The first intervention for late-life psychosis depends on a thorough diagnostic approach, as previously described. Eliminating the cause of secondary psychosis, which should be followed by appropriate management if there is a secondary etiology, including medications, neurological disorders, concomitant delirium, or superimposed condition of delirium, is the first step needed in a clinical setting. When psychotic symptoms are associated with a primary psychiatric disorder, clinicians should manage symptoms first with a non-pharmacological treatment modality, followed by pharmacological treatment when symptoms fail. Theoretically, pharmacological approaches constitute the second line of treatment for psychosis in older adults because of the adverse events commonly observed in them [21,37,41]. However, when psychotic symptoms are harmful to individuals or others, imminent management with pharmacological approaches is important. The core feature of psychotic symptoms is the problem of reality testing, which sometimes leads to a high risk of suicide after psychotic depression. 

Non-pharmacological interventions in late-life psychosis involve thorough education for patients and family, cognitive behavior therapies (CBT), family interventions to subside conflicts in caregiving, assertive community treatment, training for social skills, and supporting socioeconomic approaches to enhance treatment adherence. Continuous support for caregivers and family should be delivered simultaneously with patient-oriented interventions [42]. CBT is known for effectiveness in improving positive and negative symptoms, with a positive impact on treatment compliance [43], but still lacks evidence from older adults with psychotic disorders [44]. Other approaches to obtain clinical efficacy, including functional adaptation skills training, adaptation training with a cognitive approach, and integrated psychological therapy, involve four traditional non-pharmacological modalities; CBT, cognitive remediation therapy, family interventional therapy, and social skill training; integrating pros and cons [45]. Other interventions, as previously described, such as assertive community treatment, self-management training, lifestyle interventions, and family psychoeducation, are all reported to have a positive impact on individuals with schizophrenia, but more evidence regarding late-onset psychosis individuals is needed [46]. 

Adapted exercise programs also showed a positive impact on slowing down the decline in institutionalized older adults, especially in terms of quality of life. However, there is little evidence of positive impact on psychotic symptom control [47]. More clinical studies with a greater amount of data and longitudinal follow up are necessary to propose as thorough guidelines to managing psychotic symptoms and preventing cognitive decline in these individuals. 

Due to the risk of adverse effects, including sudden death in older adults, contemporary guidelines suggest that antipsychotics should be prescribed cautiously using a low dosage and over a short period [4]. Usually, the adverse effect profiles are coupled to specific categories of antipsychotics, but common adverse effects include sedation, which may be associated with frequent falls, anticholinergic effects, cardiovascular effects, extrapyramidal symptoms and tardive dyskinesia, metabolic effects, hyperprolactinemia, agranulocytosis, and neuroleptic malignant syndrome [48].

Typical and atypical antipsychotics have different strengths and limitations; typical antipsychotics have fewer metabolic side effects, but the risk of tardive dyskinesia is more common [49]. On the other hand, atypical antipsychotics are associated with a lower risk of tardive dyskinesia than typical antipsychotics but a higher risk of metabolic adverse effects that could threaten other mortalities. Careful assessments with the concomitant use of antipsychotics, including electrocardiography, laboratory tests, and thorough physical examinations, should be performed in clinical settings.

Due to the small sample sizes used and low prevalence of late-life psychosis, few studies have evaluated the efficacy of antipsychotics in treating psychotic symptoms of late-onset schizophrenia. Some evidence supports the use of atypical antipsychotics, especially olanzapine and risperidone, in older individuals with schizophrenia, but with careful approaches and regular check-ups for adverse events [21]. Promising results for the efficacy of long-acting injectable antipsychotics for the treatment of early-onset schizophrenia are yet to be confirmed for efficacy; the evidence is limited. These agents, including paliperidone and risperidone, have shown positive effects on delusional disorders with improvements in both positive and negative symptom profiles but lacked clinical implications for patients with an average age in their 50s. 

For the treatment of psychotic depression, tricyclic antidepressants and serotonin reuptake inhibitors have been reported to be less effective, whereas electroconvulsive treatment has shown potential for more effective symptomatic control in patients with psychosis [50]. Clinicians should consider using combined treatment with antipsychotics and antidepressants based on recent meta-analysis results showing superior clinical outcomes of psychotic depression [51].

Brain stimulation treatments, including electroconvulsive treatment (ECT) and transcranial magnetic stimulation (TMS), can also be considered in numerous psychiatric disorders, especially in treatment-resistant settings [21,52]. However, limited research is available for these modalities in older adults with psychosis. Promising results of clinical improvement in the overall psychotic symptoms were observed in middle-aged to older adults with schizophrenia, but its efficacy did not persist, with an increase in the relapse rate at six months and one year later [53]. Further research on the treatment efficacy of ECT found evidence of the effectiveness of the combination of antipsychotics and ECT for these individuals, leading towards a significantly lower risk of further relapse. However, evidence of the effects of these brain stimulation treatments on late-onset psychosis is limited due to the limited number of patients and trials. 

To build up evidence for expanding clinical characteristics and treatment modalities for late-life psychosis, there should be more trials involving larger samples, more trials with different countries working in consortia, and unified operational definitions for diagnosis. We tried to deliver clear summarized things during clinical practice in the Box 1.

Box 1 **Things to consider in individuals with late-onset psychosis, during clinical practice.**

Careful intervention:

- Higher risks of secondary psychosis;
- Higher morbidity and mortality rates;
- Higher incidence of adverse effects.

Considerations for differential diagnoses:

- Neurodegenerative disorders;
- Possibility of delirium or superimposed delirium;
- Substance-induced psychosis, including prescribed medications and illicit drugs.

Difference between early-onset schizophrenia and late-onset schizophrenia:

- More prevalent in female individuals;
- With clinical phenotypes, more common hallucinations (visual, olfactory, or tactile) and delusions (persecutory and partitioned), but less severe positive symptoms in individuals with late-onset schizophrenia.

Non-pharmacological treatments are the first option in managing late-life psychosis.

Low-dose atypical antipsychotics are frequently considered, and close observation for the possible side effects is necessary.

Thorough psychoeducation of both patient and family members is necessary to improve adherence.

## 6. Precision Medicine in Late-Onset Psychosis

As was previously discussed in a different report, precision medicine is frequently replaced with personalized medicine and individualized medicine. From this perspective, treatments and decisions would be decided by the needs of each individual on the basis of genetic, biomarker, phenotypes, or psychosocial characteristics [54]. Therefore, with these findings related to precision medicine, we could expect improved outcomes, decreased side effects with expected prognosis, and decreased morbidity and mortality [55]. 

There are numerous approaches to attain results through precision medicine in psychotic disorders, including research related to the differentiation between early-onset schizophrenia and late-onset schizophrenia based on neuroimaging findings [24], molecular research related to the age of onset of schizophrenia [56], genetic research related to psychopharmacologic response [57], and research related to the etiologies of schizophrenia [58]. However, the complexity of psychotropic drug metabolism and scarce data related to late-onset psychosis still creates limitations in applying pharmacogenetics in clinical fields; vigorous pharmacogenetic tests are needed [59]. 

Even though there are few cohorts and few genetic data related to late-onset psychotic disorders, recent research that identified and prioritized genes involved in schizophrenia reported genetic risk components for the classic age of schizophrenia onset [60]. Based on this result, they suggest a difference between those individuals with typical onset age and those with atypically early- or late-onset age. These results still need to be replicated by other cohorts or similar genetic studies, but they suggest different approaches to discover genetic backgrounds in early-onset psychosis and those in late-onset psychosis. There is a high amount of genetic research related to schizophrenia [61,62,63], but the underlying difference between schizophrenic individuals with the typical onset age and those with late-onset schizophrenia means that more investigation is needed in this field. Another approach to find the pathophysiology of late-onset psychosis targets the shared liability between late-onset psychosis and the neurodegenerative disorder accompanying psychotic symptoms. Recent genetic studies reported a shared genetic liability between schizophrenia and Alzheimer’s disease and the accompanying psychotic symptoms, suggesting a new transdiagnostic perspective on psychotic symptoms beyond the age of individuals is needed [64]. These approaches are grounded in the frequent prevalence of psychotic symptoms observed in neurodegenerative disorders and the effectiveness of atypical antipsychotics both in late-onset psychosis individuals and in those with neurodegenerative disorders showing psychotic symptoms. Research to predict the prognosis of late-life psychosis is also in progress, but needs more longitudinal data. Recent meta-analyses and longitudinal analyses have suggested APOE-ε4 as an age-related risk factor for aggressive hallucinations and delusions, and as having an age-mediated pathophysiological role in schizophrenia. This finding could support those individuals with the APOE-ε4 allele being treated with more intensive monitoring and additional interventions in mid-to-late life [65]. However, these results need to be replicated in other clinical studies. On the subject of prognosis, while pharmacological response rates and tolerability should be considered, the risk of cognitive decline, including dementia, should also be taken into account [66]. Medical conditions comorbid to late-onset psychosis could also have an impact on higher mortality rates in those individuals than in patients with early-onset psychosis, which suggests that more elaborate research on comorbid physical conditions and their clinical influence is needed [3]. 

## 7. Conclusions

Late-onset psychosis can result from disorders secondary to systemic medical disease, neurodegenerative disorders, and substance-related conditions. After thorough collateral history taking and physical examination and subsequent laboratory tests and neuroimaging, clinicians can draw etiological opinions on this condition. There are no pathognomonic signs or symptoms of late-onset psychosis for etiological diagnosis, which requires careful observation and approaches. When clinicians find the etiology of a symptom, the appropriate management of the primary diagnosis should be ensured. When a primary psychiatric disorder accounts for psychotic symptoms in older adults, with consideration of the possibility of harm to the patient or others, prompt treatment should be undertaken. Pharmacological treatment, including antipsychotics, should be carefully initiated with the lowest dosage for short-term efficacy and monitoring for adverse side effects. 

## 8. Key Messages

- Late-onset psychosis covers primary and secondary psychosis, which should be ruled out in the first clinical setting for its appropriate management.
- Careful history taking from the patient and the collateral figures is necessary.
- Laboratory tests, physical examinations, neuroimaging studies, and reviews of current medications, including over-the-counter drugs, are important, as well as a prompt approach to managing self-harm or the risk of harm to others.
- Recommendations on pharmacological treatment emphasize regular monitoring for adverse events, and a low dosage over a short period should be considered primarily in older adults.

## Data Availability

Not applicable.
